# Front vs Back and Barbell vs Machine Overhead Press: An Electromyographic Analysis and Implications For Resistance Training

**DOI:** 10.3389/fphys.2022.825880

**Published:** 2022-07-22

**Authors:** Giuseppe Coratella, Gianpaolo Tornatore, Stefano Longo, Fabio Esposito, Emiliano Cè

**Affiliations:** ^1^ Department of Biomedical Sciences for Health, Università Degli Studi di Milano, Milan, Italy; ^2^ IRCSS Galeazzi Orthopedic Institute, Milan, Italy

**Keywords:** military press, shoulder press, strength training, deltoids, pectoralis major, EMG, exercise, fitness

## Abstract

Overhead press is commonly performed to reinforce the muscles surrounding the shoulders. However, many overhead press variations can be executed, thus varying the stimuli to each muscle. Therefore, the current study compared the muscles excitation during overhead press performed with the barbell passing in front or behind the head or using a shoulder press machine. Eight competitive bodybuilders performed in random order front (front-BMP) or back barbell military press (back-BMP), and front (front-MSP) with neutral handgrip or back machine shoulder press (back-MSP). Normalized surface electromyographic root mean square (RMS) of anterior, medial and posterior deltoid, upper trapezius, pectoralis major and triceps brachii was recorded during both the ascending and descending phases. During the ascending phase, anterior deltoid showed greater RMS in back-BMP than back-MSP [ES: 1.42, (95% confidence interval 0.32/2.51)]. Medial deltoid showed greater RMS in back-BMP than front-BMP [ES: 3.68 (2.07/5.29)], and back-MSP [ES: 7.51 (4.73/10.29)]. Posterior deltoid showed greater RMS in back-BMP than front-BMP [ES: 9.00 (5.73/12.27)]. Pectoralis major showed greater RMS in front-BMP than back-BMP [ES: 3.11 (1.65–4.56)] and in front-MSP than back-MSP [ES: 20.52 (13.34/27.70)]. During the descending phase, anterior deltoid was more excited in back-BMP compared to front-BMP [ES: 7.66 (4.83/10.49). Medial deltoid showed greater RMS in back-BMP than front-BMP [ES: 4.56 (2.70/6.42)]. Posterior deltoid showed greater RMS in back-BMP than front-BMP [ES: 8.65 (5.50/11.80)]. Pectoralis major showed greater RMS in front-BMP than back-BMP [ES: 4.20 (2.44/5.95)]. No between-exercise difference was observed for upper trapezius. Performing back overhead press enhances the excitation of medial and posterior and partly anterior deltoid, while front overhead favors pectoralis major. Overhead press performed using barbell excites muscles more than using machine to stabilize the trajectory of the external load. Different variations of overhead press appear to provide different stimuli to the shoulder muscles and may be used accordingly during the training routine.

## Introduction

Resistance training is widely used to increase muscle strength and induce structural adaptations (Coratella and Schena, 2016; Schoenfeld et al., 2016). Since each exercise provides a unique mechanical stimulus to the targeted muscles and encompasses a unique neural pattern, understanding how each exercise excites (Vigotsky et al., 2018) the muscles involved could help to choose the exercises within a given session depending on the aims. When the purpose is to stimulate the muscles surrounding the shoulders, overhead press is one of the most used multi-joint exercises (Ichihashi et al., 2014; McKean and Burkett, 2015; Williams et al., 2020). Particularly, the simultaneous scapular upward rotation (Ichihashi et al., 2014), together with the humerus abduction and elbow extension (Paoli et al., 2010; Saeterbakken and Fimland, 2013) makes the overhead press suitable to stimulate upper trapezius, deltoids and triceps brachii.

When performing overhead press with a barbell, the barbell can pass in front or behind the head, resulting in front (front-BMP) or back barbell military press (back-BMP). While a number of studies have examined the muscles excitation during front-BMP (Kohler et al., 2010; Saeterbakken and Fimland, 2013; Williams et al., 2020), no information are currently available on the muscles excitation during back-BMP, and a direct comparison is consequently not even available. This might derive from a possible fear to perform back-BMP, maybe related to the “high-five” position that might be associated with a greater prevalence anterior shoulder instability in amateurs, albeit these results are not conclusive and should be interpreted with caution (Kolber et al., 2013). However, it was reported that both front- and back-BMP are safe exercises for participants with normal trunk stability and ideal shoulder ROM (McKean and Burkett, 2015), and still no study has compared the muscles excitation in the two so far. Moreover, overhead press can also be performed using a machine shoulder press (MSP). Most shoulder press have a forward and a lateral grip, so front-MSP and back-MSP can be performed, simulating the barbell’s trajectory. However, although the machine levers mimic the barbell’s trajectory, the barbell presents a trajectory closer to a translation and the machine rotates around an axis in the equipment; additionally, the hands end the movement much closer in MSP than BMP, and all these factors result in different movements that could affect the muscle excitation. Also in this case, no information concerning the muscle excitation during front-MSP and back-MSP has been collected to date. However, the diversification of both neuromuscular and mechanical stimuli over consecutive mesocycles is an important factor in resistance training, so that the possible between-variation differences should be acknowledged to choose one or another in the training practice.

When investigating the muscles excitation in resistance exercises, the examination should describe separately the ascending from the descending phase. Indeed, the unique neural activation of the concentric vs. eccentric action (Duchateau and Enoka, 2016), as well as the acute (Beato et al., 2021), short-term (Coratella and Bertinato, 2015; Coratella et al., 2016) and long-term (Coratella et al., 2015; Coratella et al., 2019) distinct characteristics of the eccentric phase may provide useful information to the trainers for appropriately selecting each exercise. Lastly, it was suggested that bodybuilders exhibit greater control of individual muscles when practicing their training routines (Maeo et al., 2013), and may suit for describing each exercise with a consistent technique. Therefore, the present study aimed to investigate the between-exercise differences in deltoids, pectoralis major, upper trapezius and triceps brachii excitation comparing front-BMP, back-BMP, front-MSP and back-MSP. The exercises were performed by competitive bodybuilders, and both the ascending and the descending phases were separately examined. It was hypothesized that back exercises would excite more the posterior deltoid, while the management of the barbell trajectory compared to the fixed trajectory of the machine’s lever would require more muscle excitation for stabilization purposes.

## Materials and Methods

### Study Design

The present investigation was designed as a cross-over, repeated-measures, within-subject study. The participants were involved in seven different sessions. In the first session, the participant were familiarized with the technique of each exercise. From session two to five, the 1-RM was measured in front-BMP, back-BMP, front-MSP or back MSP in random order. In the sixth session, the participants were familiarized with the selected loads and the electrodes placement. In the seventh session, the muscles’ maximum excitation was first measured. Then, after a minimum of 30 min of passive recovery, the participants performed a non-exhausting set for each exercise in a random order, with an inter-set pause of 10 min. Each session was separated by at least 3 days, and the participants were instructed to avoid any further form of resistance training for the entire duration of the investigation.

### Participants

The present investigation was advertised by the investigators during some regional and national competitions, and to be included in the study, the participants had to compete in regional competitions for a minimum of 5 years. Additionally, they had to be clinically healthy, without any reported history of upper-limb and lower back muscle injury and neurological or cardiovascular disease in the previous 12 months. To avoid possible confounding factors, the participants competed in the same weight category (Men’s Classic Bodybuilding <80 kg, <1.70 m), according to the International Federation of Body Building Pro-League. The use of drugs or steroids was continuously monitored by a dedicated authority under its regulations, although we could have not checked for it. Thereafter, a convenience sample of eight male competitive bodybuilders (age 29.8 ± 3.0 years; body mass 77.9 ± 1.0 kg; stature 1.68 ± 0.01 m; training seniority 10.6 ± 1.8 years) were recruited for the present procedures, in line with previous studies (Coratella et al., 2020b; Coratella et al., 2020c; Coratella et al., 2021; Coratella et al., 2022). The participants were asked to abstain from alcohol, caffeine, or similar beverages in the 24 h preceding the test. After a full explanation of the aims of the study and the experimental procedures, the participants signed a written informed consent. They were also free to withdraw at any time. The current design was approved by the Ethical Committee of the Università degli Studi di Milano (CE 27/17) and performed following the Declaration of Helsinki (1975 and updates) for studies involving human subjects. The individual in this manuscript has given written informed consent to publish these case details.

### Exercises Technique

**FIGURE 1:**
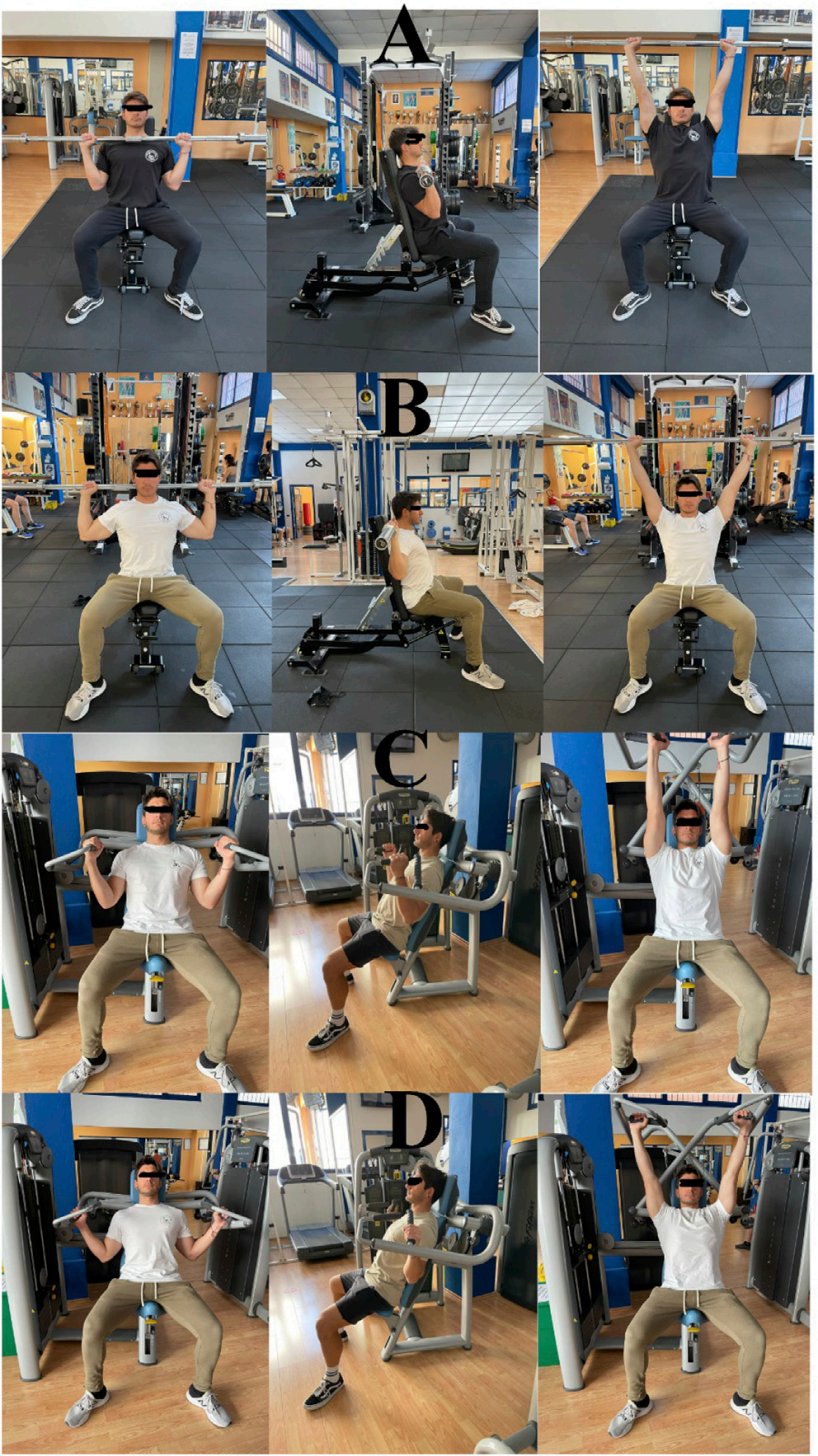
The technique for each exercise, described with a frontal and lateral view of the start and a frontal view of the end of each movement: **(A)** front barbell military press; **(B)** back barbell military press; **(C)** front machine shoulder press; **(D)** back machine shoulder press.

The front- and back-BMP were performed seated on a bench (Technogym, Cesena, Italy), with the backseat inclined at 80° to stabilize the trunk. The barbell was an Olympic model (Vulcan Standard 20 kg, Vulcan Strength Training System, Charlotte, NC, United States). The front-BMP was performed holding the barbell a handbreadth outside the deltoids, and the participants were required to lift the barbell from the upper chest up to straight the elbow above and behind the head. Additionally, they were instructed to maintain the forearm perpendicular to the ground throughout the whole movement, and the movement was checked visually by an operator. Consequently, the barbell’s trajectory in front-BMP was not straight but resulted in a more curvilinear C-shape trajectory. The back-BMP was performed holding the bar at an inter-hand distance so that at 90° shoulder/humerus joint corresponded 90° at the elbow (Figure 1). The participants were instructed to lift the barbell starting from below the external occipital protuberance up to a full elbow extension (Paoli et al., 2010), passing behind the head and resulting in an approximately straight trajectory. During the execution of the back-BMP, the trunk was inclined at 90° to let the bar passing behind the neck, but still having a lumbar support.

The front- and back-MSP were performed using a dynamic-constant external load device (Shoulder press, Technogym, Cesena, Italy). The displacement of the external resistance (i.e., barbell or machine’s lever) was roughly similar for each corresponding variation, i.e., front-BMP vs. front-MSP and back-BMP vs. back-MSP, meaning that the start and the end of the movement resulted in comparable movements, as visually checked. The height of the seat was tailored depending on the participant’s stature, so to have the start of the ascending phase above the no-load position. The front-MSP was performed holding the forward-neutral grip as designed by the manufacturer (Figure 1) and the back-MSP holding the lateral-prone grip, at the same between-hand distance of back-BMP.

For each exercise, the time under tension was 2 s for the ascending and descending phase, with an isometric phase lasting approximately 0.5 s, and a visual time-feedback was provided (Coratella et al., 2020a; Coratella et al., 2020b; Coratella et al., 2020c; Coratella et al., 2021). After a warm-up consisting of 2 × 15 repetitions at a self-selected load, the participants performed six repetitions at 80% 1-RM to avoid fatigue. To this purpose, at the end of the 10 min inter-set recovery each participant was asked if he would have been able to perform the subsequent set, and in case of negative response more time was provided. The set was repeated in case of disproportionate duration of any phase.

### 1-RM Protocol

The 1-RM was assessed using the same exercises’ technique described above. Briefly, after a standardized warm-up consisting of 3 × 10 repetitions of the tested exercise using three incremental self-selected loads, the 1-RM attempts started from the 80% of the self-declared 1-RM and additional 5% or less was added until failure to lift the load (Coratella and Schena, 2016). Each attempt consisted in one repetition and was separated by at least 3 min of passive recovery. Strong standardized encouragements were provided to the participants to maximally perform each trial.

### Maximum Voluntary Isometric Excitation

The maximal voluntary isometric excitation of anterior deltoid, medial deltoid, posterior deltoid, the clavicular head of pectoralis major, upper trapezius and the lateral head of triceps brachii was measured in random order following the SENIAM (surface electromyography for the non-invasive assessment of muscles) procedures (Hermens et al., 2000). The electrodes (mod H124SG Kendall ARBO; diameter: 10 mm; inter-electrodes distance: 20 mm; Kendall, Donau, Germany) placement was in line with the SENIAM recommendations (Hermens et al., 2000). The electrodes were equipped with a probe (probe mass: 8.5 g, BTS Inc., Milano, Italy) that permitted the detection and the transfer of the surface electromyography (sEMG) signal by wireless modality. The sEMG signal was acquired at 1,000 Hz, amplified (gain: 2,000, impedance and the common rejection mode ratio of the equipment are >1,015 Ω//0.2 pF and 60/10 Hz 92 dB, respectively) and driven to a wireless electromyographic system (FREEEMG 300, BTS Inc., Milano, Italy) that digitized (1,000 Hz) and filtered (filter type: IV-order Butterworth filter, band-pass 10–500 Hz) the raw sEMG signals.

The sEMG electrodes for the anterior deltoid were placed over the mid-belly of the muscle approximately 4 cm below the clavicle (Hermens et al., 2000). The participants were then instructed to flex the elbow to 90° so that the hand was pointed upwards and asked to make a closed fist with the hand of the flexed arm and to provide maximal force to produce shoulder flexion against manual resistance (Coratella et al., 2020b). The electrodes of the medial deltoid were placed on the lateral aspect of the deltoid, 3 cm below the acromion process (Hermens et al., 2000). The participants were then instructed to flex the elbow to 90° and were asked to maximally abduct the flexed arm against manual resistance (Coratella et al., 2020b). For posterior deltoid, the electrodes were placed in the area about two fingerbreadths behind the angle of the acromion (Hermens et al., 2000). The participants were asked to abduct the shoulder in a slight extension against manual resistance, with the humerus in slight internal rotation (Coratella et al., 2020b). The sEMG electrodes for the clavicular head of the pectoralis major were placed on the midclavicular line, midway between the acromioclavicular joint of the shoulder for the clavicular head (Hermens et al., 2000). The participants were instructed to horizontally abduct the arm with the shoulder and elbow flexed at 90 and to provide maximal force while attempting to horizontally adduct the arm against unmovable resistance (Coratella et al., 2020b; Coratella et al., 2020c). For upper trapezius, the electrodes were placed at 50% on the line from the acromion to the spine on vertebra C7 (Hermens et al., 2000). The participants were instructed to elevate the acromial end of the clavicula and scapula against unmovable resistance pushing downward (Coratella et al., 2020b). For the lateral head of triceps brachii, the electrodes were placed at 50% on the line between the posterior crista of the acromion and the olecranon at two finger widths lateral to the line (Hermens et al., 2000). The participants were instructed to extend the elbow against unmovable resistance toward the elbow flexion (Coratella et al., 2020b). Each attempt lasted 5 s, and three attempts were completed for each movement interspersed by 3 min of passive recovery (Coratella et al., 2020b; Coratella et al., 2020c). The operators provided strong standardized verbal encouragements. In line with previous procedures, the electrodes were placed on the dominant limb (Coratella et al., 2020a; Coratella et al., 2020c; Coratella et al., 2021).

**FIGURE 2:**
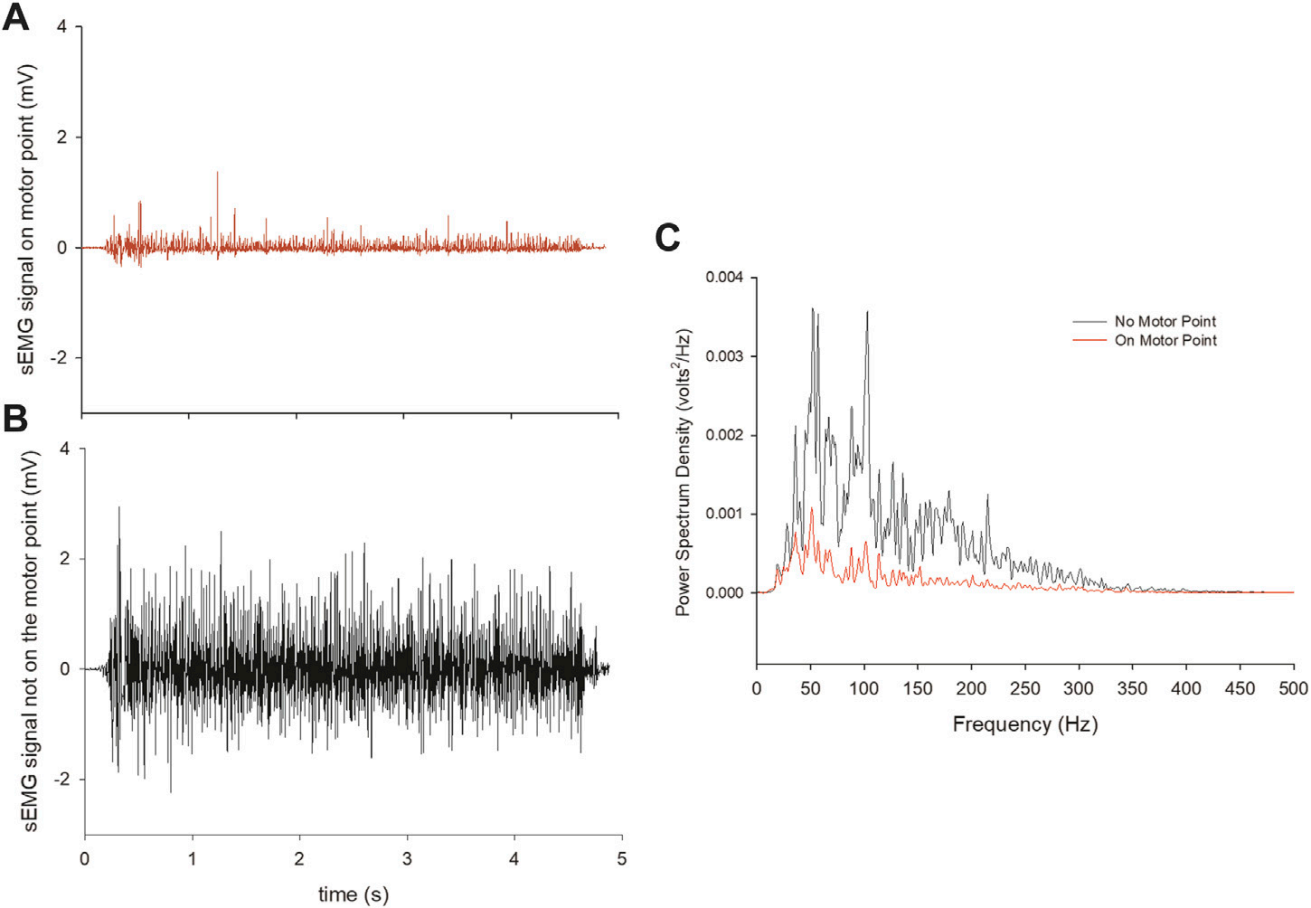
An example from a representative participant of the sEMG signal deriving from an appropriate **(A)** or non-appropriate **(B)** electrode placement. The power spectrum density is also shown for the two cases **(C)**.

To check for appropriate electrodes placement previous procedures were followed (Coratella et al., 2020a; Coratella et al., 2020b; Coratella et al., 2020c; Coratella et al., 2021). For example, if the electrode shifted over the innervation zone during part of the movement, the EMG amplitude was underestimated. Therefore, to check for any consequence due to a possible shift of the surface electrode over the innervation zone, a Fast-Fourier Transform approach was used, as suggested in a previous investigation (Merlo and Campanini, 2010). Briefly, the electrode placement on each muscle was checked during the warm-up phase of each exercise, analysing the power spectrum profile of the sEMG signal recorded at the starting-, middle-, and end-point of each exercise in all muscles. The correct electrode placement results in a typical belly-shaped power spectrum profile of the EMG signal, while noise, motion artifacts, power lines, and electrodes placed on the innervation zone or myotendinous junction generate a different power spectrum profile (Merlo and Campanini, 2010). If the power spectrum did not match with the typical belly-shaped power spectrum profile in any of the temporal points, the electrodes were repositioned, and the procedures repeated so to have a clear EMG signal from all the muscles throughout the movement. Figure 2 shows two representative cases where the electrodes were placed in the correct or non-appropriate position, and the different sEMG signal resulting from both. The same experienced operator placed the electrodes and checked the power-spectrum profile. This approach was shown to provide very high inter-session reliability in sEMG data for these muscles (Coratella et al., 2020b; Coratella et al., 2020c).

### Data Analysis

The sEMG signals from both the peak value recorded during the maximum voluntary isometric activation and from the ascending and descending phases of each exercise were analysed in time-domain, using a 25-ms mobile window for the computation of the root mean square (RMS). For the maximum voluntary isometric activation, the average of the RMS corresponding to the central 2 s was considered. During each exercise, the RMS was calculated and averaged over the 2 s of the ascending and descending phase. To identify the ascending and the descending phase, the sEMG was synchronized with an integrated camera (VixtaCam 30 Hz, BTS Inc., Milano, Italy) that provided the duration of each phase (Coratella et al., 2020a; Coratella et al., 2020b; Coratella et al., 2021). Such a duration was used to mark the start and the end of each phase while analysing the sEMG signal. The sEMG data were averaged excluding the first and the last repetition of each set, to possibly have more consistent technique and decreasing the interference of fatigue. After, the sEMG RMS of each muscle during each exercise was normalized (nRMS) for its respective maximum voluntary isometric excitation (Coratella et al., 2020a; Coratella et al., 2020b; Coratella et al., 2020c; Coratella et al., 2021) and inserted into the data analysis.

### Statistical Analysis

The statistical analysis was performed using a statistical software (SPSS 22.0, IBM, Armonk, NY, United States). The normality of data was checked using the Shapiro–Wilk test and all distributions were normal (*p* > 0.05). Descriptive statistics (participants = 8) are reported as mean (SD). The differences in the nRMS were separately calculated for each exercise considering the type of external load (barbell or machine: 2 levels), the position of external load (front vs. back: 2 levels), and phase of the exercise (ascending or descending: 2 levels) using a three-way repeated-measures ANOVA. Multiple comparisons were adjusted using the Bonferroni’s correction. Significance was set at α < 0.05. The magnitude of the interactions and single factors was calculated using partial eta squared ( η _p_^2^). The pairwise differences are reported as mean Cohen’s d effect size (ES) with 95% confidence interval (95% CI), and ES was interpreted according to the Hopkins’ recommendations: 0.00–0.19: trivial; 0.20–0.59: small: 0.60–1.19: moderate; 1.20–1.99: large; ≥2.00: very large (Hopkins et al., 2009).

## Results

The 1-RM was 82 (8) kg for front-BMP, 76 (7) kg for back-BMP, 95 (9) kg for front-MSP and 87 (8) for back-MSP.

**FIGURE 3:**
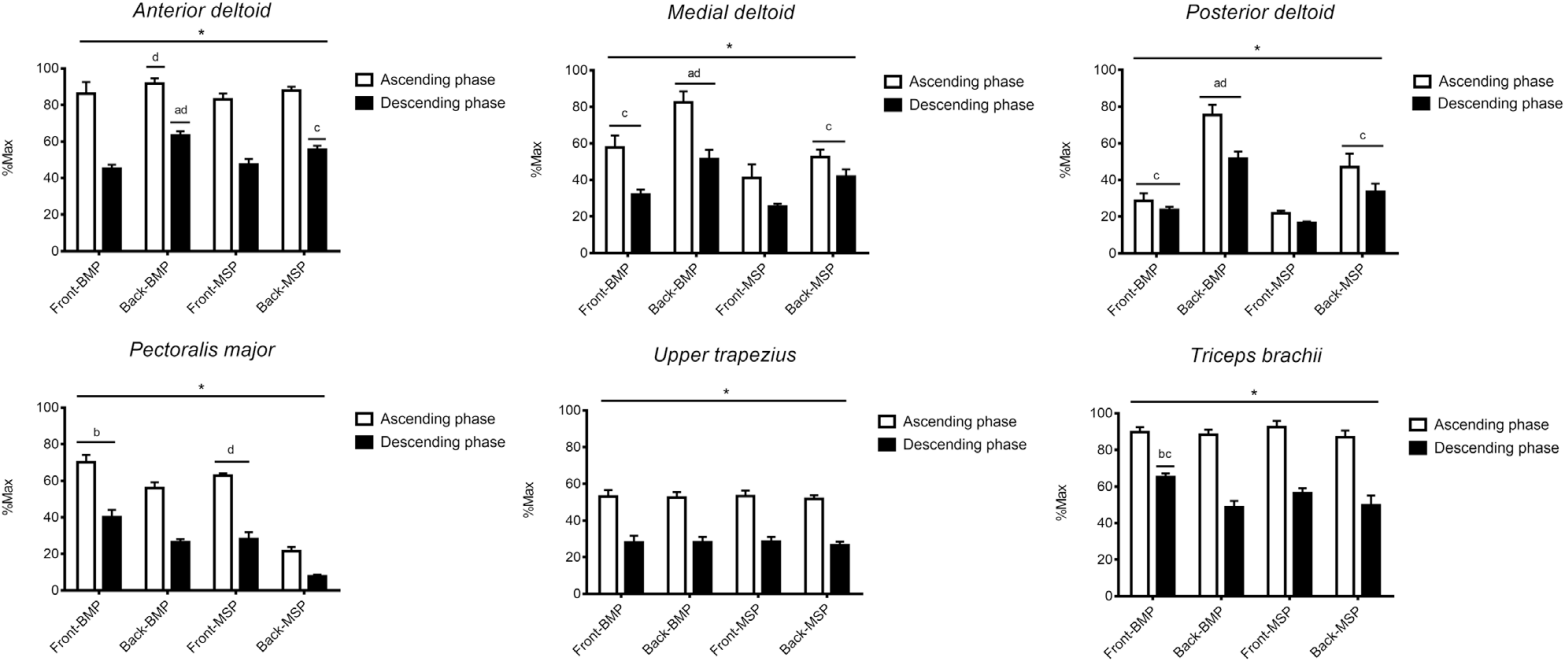
Mean (SD) for the sEMG RMS of the muscles examined during the ascending and descending phase of each exercise. Besides the front vs. back and barbell vs. machine differences, sEMG RMS was greater during the ascending than descending phase in all exercises. a: *p* < 0.05 vs. front-BMP. b: *p* < 0.05 vs. back-BMP. c: *p* < 0.05 vs. front-MSP. d: *p* < 0.05 vs. back-MSP.

Figure 3 shows the nRMS recorded in all muscles during the two phases of the four exercises. Load × position × phase interaction (F_1,7_ = 9.950, *p* = 0.016, η _p_^2^ = 0.587) was found for the nRMS of anterior deltoid. Additionally, load × position (F_1,7_ = 12.679, *p* = 0.009, η _p_^2^ = 0.644), position × phase (F_1,7_ = 18.561, *p* = 0.004, η _p_^2^ = 0.726), but not load × phase interaction (F_1,7_ = 0.168, *p* = 0.694, η _p_^2^ = 0.023) was observed, and main effect for factor load (F_1,7_ = 16.189, *p* = 0.020, η _p_^2^ = 0.561), position (F_1,7_ = 54.433, *p* < 0.001, η _p_^2^ = 0.937), and phase (F_1,7_ = 98.572, *p* < 0.001, η _p_^2^ = 0.988) was found. During the ascending phase, greater nRMS was found in back-BMP than front-MSP [ES: 2.80 (95% confidence interval 1.42/4.18)] and back-MSP [ES: 1.42 (0.32/2.51)]. During the descending phase, anterior deltoid was more excited in back-BMP compared to front-BMP [ES: 7.66 (4.83/10.49) and back-MSP [ES: 3.26 (1.76/4.75)]. Additionally, greater excitation was found in back-MSP than front-MSP [ES: 2.86 (1.47/4.25)]. The nRMS was greater during the ascending vs. descending phase in all exercises (ES ranging from 4.83 to 12.38).

Load × position × phase (F_1,7_ = 7.716, *p* = 0.027, η _p_^2^ = 0.524), load × position (F_1,7_ = 12.198, *p* = 0.010, η _p_^2^ = 0.635), load × phase (F_1,7_ = 51.289, *p* < 0.001, η _p_^2^ = 0.880), but not position × phase interaction (F_1,7_ = 0.001, *p* = 0.985, η _p_^2^ = 0.001) was found for the nRMS of medial deltoid, with main effect observed for factor load (F_1,7_ = 84.543, *p* < 0.001, η _p_^2^ = 0.967), position (F_1,7_ = 86.691, *p* < 0.001, η _p_^2^ = 0.969) and phase (F_1,7_ = 77.194, *p* < 0.001, η _p_^2^ = 0.959). During the ascending phase, greater nRMS was found in back-BMP than front-BMP [ES: 3.68 (2.07/5.29)] and back-MSP [ES: 7.51 (4.73/10.29)]. Moreover, front-BMP showed greater nRMS than front-MSP [ES: 2.25 (1.00/3.50)], and back-MSP showed greater RMS than front-MSP [ES: 1.81 (0.65/2.98)]. During the descending phase, greater nRMS was found in back-BMP than front-BMP [ES: 4.56 (2.70/6.42)] and back-MSP [ES: 2.03 (0.82/3.24]. Additionally, back-MSP showed greater nRMS than front-MSP [ES: 5.16 (3.12/7.20)], and front-BMP showed greater nRMS than front-MSP [ES: 2.78 (1.40/4.15)]. The RMS was greater during the ascending vs. descending phase for all exercises (ES ranging from 5.12 to 7.55).

Load × position × phase (F_1,7_ = 8.100, *p* = 0.025, η _p_^2^ = 0.536), load × position (F_1,7_ = 97.288, *p* < 0.001, η _p_^2^ = 0.933), load × phase (F_1,7_ = 5.892, *p* = 0.046, η _p_^2^ = 0.457) and position × phase interaction (F_1,7_ = 27.824, *p* = 0.001, η _p_^2^ = 0.799) was found for the nRMS of posterior deltoid, with main effect observed for factor load (F_1,7_ = 93.917, *p* < 0.001, η _p_^2^ = 0.975), position (F_1,7_ = 96.736, *p* < 0.001, η _p_^2^ = 0.991) and phase (F_1,7_ = 80.794, *p* < 0.001, η _p_^2^ = 0.920). During the ascending phase, greater nRMS was observed in back-BMP than front-BMP [ES: 9.00 (5.73/12.27)] and back-MSP [ES: 4.09 (2.37/5.82)]. Moreover, back-MSP showed greater nRMS than front-MSP [ES: 4.42 (2.60/6.23)], and front-BMP showed greater nRMS than front-MSP [ES: 2.08 (0.87/3.30)]. During the descending phase, greater nRMS was observed in back-BMP than front-BMP [ES: 8.65 (5.50/11.80)] and back-MSP [ES: 4.04 (2.33/5.75)]. Additionally, back-MSP showed greater nRMS than front-MSP [ES: 5.03 (3.03/7.03)], and front-BMP showed greater nRMS than front-MSP [ES: 4.86 (2.91/6.81)]. The nRMS was greater during the ascending vs. descending phase for all exercises (ES ranging from 2.67 to 10.51).

No load × position × phase interaction (F_1,7_ = 0.237, *p* = 0.869, η _p_^2^ = 0.111) was found for the nRMS of upper trapezius, albeit the main effect observed for factor phase (F_1,7_ = 93.901, *p* < 0.001, η _p_^2^ = 0.977). No between-exercise difference was observed during both the ascending and descending phase. The nRMS was greater during the ascending vs. descending phase for all exercises (ES ranging from 3.60 to 4.34).

Load × position × phase (F_1,7_ = 53.082, *p* < 0.001, η _p_^2^ = 0.883), load × position (F_1,7_ = 109.342, *p* < 0.001, η _p_^2^ = 0.970), load × phase (F_1,7_ = 10.540, *p* = 0.014, η _p_^2^ = 0.601) and position × phase interaction (F_1,7_ = 58.940, *p* < 0.001, η _p_^2^ = 0.894) was found for the nRMS of pectoralis maior, with main effect observed for factor load (F_1,7_ = 154.915, *p* < 0.001, η _p_^2^ = 0.995), position (F_1,7_ = 149.476, *p* < 0.001, η _p_^2^ = 0.994) and phase (F_1,7_ = 143.434, *p* < 0.001, η _p_^2^ = 0.993). During the ascending phase, greater nRMS was observed in front-BMP than back-BMP [ES: 3.11 (1.65–4.56)], and in front-MSP than back-MSP [ES: 20.52 (13.34/27.70)]. During the descending phase, greater nRMS was observed in front-BMP than back-BMP [ES: 4.20 (2.44/5.95)], and in front-MSP than back-MSP [ES: 7.03 (4.40/9.65)]. The nRMS was greater during the ascending vs. descending phase for all exercises (ES ranging from 6.25 to 21.36).

Load × position × phase interaction (F_1,7_ = 21.529, *p* = 0.002, η _p_^2^ = 0.755) was found for the nRMS of triceps brachii. Additionally, load × phase (F_1,7_ = 20.960, *p* = 0.003, η _p_^2^ = 0.750), position × phase (F_1,7_ = 24.818, *p* = 0.002, η _p_^2^ = 0.780), but not load × position (F_1,7_ = 0.492, *p* = 0.506, η _p_^2^ = 0.066) was observed, and main effect for factor position (F_1,7_ = 116.834, *p* < 0.001, η _p_^2^ = 0.943), phase (F_1,7_ = 98.572, *p* < 0.001, η _p_^2^ = 0.988), but not load (F_1,7_ = 1.671, *p* = 0.237, η _p_^2^ = 0.193) was found. During the ascending phase, no between-exercise difference was observed. During the descending phase, greater nRMS was found in front-BMP than back-BMP [ES: 8.29 (5.33/10.56)] and front-MSP [ES: 3.48 (1.97/4.68)]. Moreover, front-MSP showed greater nRMS than back-MSP [ES: 1.55 (0.49/2.47)]. The nRMS was greater during the ascending vs. descending phase for all exercises (ES ranging from 4.43 to 10.51).

## Discussion

The present study examined for the first time the excitation of the prime movers in military and shoulder press performed in front or behind the head. Differences in muscle excitation have been found between the overhead press variations analysed here, except for the upper trapezius that did not show any between-exercise dissimilarity. Comparing front vs. back, as previously hypothesized, medial and posterior deltoids were more excited in back exercises and pectoralis major in front exercises during the ascending phase. The descending phase had similar pattern, with the adjunct of anterior deltoid more excited in back and triceps brachii in front exercises. Comparing barbell vs. machine overhead press, greater muscle excitation was overall observed using barbell, in line with the initial hypothesis. Moreover, the ascending resulted in overall greater muscle excitation compared to the descending phase. The present results highlight that front and back overhead press have different muscle excitation and using barbell overall increased muscle excitation to stabilize the external load along its trajectory. In this light, the present overhead press variations should not be intended as equivalent and could be used to stimulate the muscles surrounding the shoulders differently.

### Front vs. Back

The front vs. back comparison offers interesting perspectives in term of muscle excitation. However, beside the differences in muscle excitation induced by the exercises, it should be reminded that the different between-hand distance and the possible different trajectory (C-shape for the front- and straight for the back-BMP) may affect the sEMG signal amplitude because of the muscles acting at different length (Vigotsky et al., 2018). Back-BMP showed greater excitation of posterior deltoid compared to both front-BMP and front-MSP. The greater excitation of posterior deltoid in back-BMP has similar pattern during both the ascending and descending phase compared with the remaining exercises. Posterior deltoid acts as external rotator of the humerus (Escamilla et al., 2009), and such an external rotation is needed to avoid a forward unbalance of the barbell, stabilizing its trajectory behind the head. The excitation of the posterior deltoid is a combination of both the external humerus rotation and abduction, albeit in the latter posterior deltoid is not effective as anterior and medial deltoid (Escamilla et al., 2009). However, both the high-load used here and the full range of motion may have contributed to enhance its excitation within all-exercises (Paoli et al., 2010). Notwithstanding, the back trajectory appears more suitable for exciting posterior deltoid because of the need to stabilize the barbell behind the head.

When comparing the front vs. back press, the present results suggest that back-BMP is more effective than front-BMP to excite medial deltoid, possibly deriving from the more prominent humerus abduction compared with the combined abduction-flexion performed in front-BMP. The same pattern of the ascending phase was overall maintained during the descending phase. Medial deltoid is a strong abductor of the humerus, especially when the abduction starts beyond a minimal angle (Escamilla et al., 2009). In support of the present results, a lateral glenohumeral raise was reported to excite the medial deltoid more than a frontal raise performed with similar relative load (Coratella et al., 2020b). Additionally, both the load and the full movement used here may have led to a relative high excitation of the medial deltoid (Paoli et al., 2010). Hence, an abduction performed on the lateral plane is more effective than a spurious abduction to enhance the excitation of the medial deltoid.

Given the differences in posterior and medial deltoid excitation possibly due to the degree of external rotation combined with the abduction of humerus, it may be surprising at a first glance that the anterior deltoid was strongly and similarly excited in back exercises during the ascending phase, as well as more excited in back-BMP during the descending phase. However, while it is known that the excitation of the anterior deltoid increases with along the verticality of the lifting plane (Luczak et al., 2013), anterior deltoid is a strong abductor of the humerus whatever the lifting plane (Escamilla et al., 2009), and this may partially explain the lack of difference comparing front vs. back press during the ascending phase. Moreover, the possibly more frontal lifting trajectory in front vs. back exercises that suggest greater anterior deltoid excitation may have been compensated by the greater external rotation of the humerus when performing back exercises. This was shown indeed to put anterior deltoid in a more optimal lever (Escamilla et al., 2009), inducing an increase in its excitation (Coratella et al., 2020b). Interestingly, the descending phase resulted in greater anterior deltoid excitation during back than front exercises, possibly leading to argue that the needs for maintaining an external rotated trajectory may have increased its excitation when controlling the movement, as shown when comparing the lateral raises with external vs. internal rotated humerus (Coratella et al., 2020b).

While the combined anterior flexion in front exercises is somehow compensated by the external rotation of the humerus in terms of excitation of the anterior deltoid, pectoralis major was more excited in front-BMP and front-MSP compared to back-BMP and back-MSP. This phenomenon was also observed when comparing frontal vs. lateral glenohumeral raises (Coratella et al., 2020b). Moreover, it should be noticed that front-BMP leads to a more extended thoracic trunk compared to back-BMP (McKean and Burkett, 2015), as also occurred here given the different trunk inclination (adherent to the cushion during front-BMP vs. 90° during back-BMP). This possibly implies a less vertical humerus flexion, thus requiring the pectoralis major to be more excited during front-BMP, and concurrently the deltoids more excited during back-BMP. Concerning the excitation of triceps brachii, the front vs. back press resulted in greater excitation during the descending phase. Interestingly, the front exercises lead to a more flexed humerus, thus increasing the elongation of the triceps brachii, and this was shown to positively affect the triceps excitation (Alves et al., 2018), possibly explain the results. However, the triceps excitation was close to the maximum in all exercises. Lastly, upper trapezius showed no difference when comparing front vs. back exercises. Since the scapular elevation needed to allow the humerus abduction (Escamilla et al., 2009) could be potentially similar in all variations, such a lack of difference may be expected.

### Barbell vs. Machine

As for the front vs. back comparison, we should remind that the trajectories of the overhead press performed using the barbell or the machine implies some points that should be highlighted and that could have had repercussions per se on the muscle excitation. Indeed, since the barbell is supposed to present a trajectory closer to a translation and the machine rotates around an axis in the equipment, differences in the trajectory are intrinsic in the movements (although not directly measured here), as for example concerns the back BMP vs. back MSP. Additionally, the hands end their movement much closer with the machine than the barbell, so the resulting range of movements of glenohumeral and thoracic-scapula joints are possibly intrinsically different. Together, this may lead to the muscles involved in different muscle length, feasibly affecting per se the sEMG signal amplitude (Vigotsky et al., 2018). Moreover, performing overhead press using barbell vs. machine may imply greater need for stabilization of the vertical trajectory of the load. In the present study, the posterior deltoid was overall more excited during barbell vs. machine overhead press during both the ascending and descending phase. Besides the greater stability offered by the machine press, back-BMP results in still higher excitation of the posterior deltoid than back-MSP, possibly also due to the straighter trajectory of the barbell compared to the machine’s lever. Although no previous study has directly compared the present overhead press variations, previous papers examined the muscle excitation when different level of stabilization was required. For example, in line with the present results, posterior deltoid was more excited when shoulder press was performed standing vs. seated, irrespectively if performed with barbell or dumbbells (Saeterbakken and Fimland, 2013), or barbell vs. machine exercises (McCaw and Friday, 1994; Schwanbeck et al., 2009). A similar pattern was observed here for medial deltoid, so that less stable trajectories require more excitation. In line, the same study also reported greater excitation of medial deltoid in standing vs. seated and dumbbells vs. barbell shoulder press in standing position (Saeterbakken and Fimland, 2013). In contrast, medial deltoid similarly excited during barbell vs. dumbbell overhead press performed in both stable and unstable surface (Kohler et al., 2010). Such a difference could derive from the different load used, different study design, as well as the different experience in strength training of the participants involved in the studies. Interestingly, anterior deltoid was roughly similarly excited in all exercises, except for the greater excitation observed in back-BMP vs. back-MSP during the ascending phase. The literature reports no difference in anterior deltoid excitation while performing overhead press with barbell vs. dumbbells, on both stable and unstable surfaces (Kohler et al., 2010). Similarly, no difference was observed when comparing overhead press on stable vs. unstable surface (Uribe et al., 2010), or stable vs. unstable load using barbell (Williams et al., 2020). In contrast, greater excitation was observed when performing overhead press with dumbbells vs. barbell, but not comparing seated vs. standing position (Saeterbakken and Fimland, 2013). Interestingly, a more forward and stable trajectory using dumbbells led to greater excitation of the anterior deltoid compared with a more backward and unstable press using kettlebells (Dicus et al., 2018). It should be noted that, although both barbell and machine presses were performed seated, it is possible that the participants experienced more degrees of freedom in the trunk, thus influencing the need to rotate externally the humerus. To summarize, posterior and medial deltoid appear to act as stabilizers of the load’s trajectory more than anterior deltoid, whose role was more evident in stabilizing the back-BMP.

The excitation of pectoralis major and upper trapezius did not change across the overhead press performed using barbell or machine. The role of both muscles in overhead press is to stabilize the initial phase of the movement (pectoralis major) (Dicus et al., 2018), and allow stable glenohumeral elevation by elevating the scapulae (upper trapezius) (Escamilla et al., 2009). In line, the excitation of pectoralis major did not change during overhead press performed using dumbbells vs. kettlebells (Dicus et al., 2018), or stable vs. unstable surface (Uribe et al., 2010; Williams et al., 2020). Similarly, upper trapezius was shown to excite similarly during overhead performed with stable vs. unstable load (Kohler et al., 2010; Williams et al., 2020), or on stable vs. unstable surface (Kohler et al., 2010). Greater excitation for the triceps brachii during the descending phase was found in front-BMP vs. front-MSP, indicating that more control is needed when accompanying a more unstable load towards the lowest position. The comparison with the literature may suffer from two main concerns: first, the head selected as representative of the triceps muscle (lateral, medial or long), and second, the lack of distinction between the ascending and descending phase. Stable surface and load elicited more excitation of triceps brachii (Kohler et al., 2010) while a recent study did not find any difference when comparing stable vs. unstable load, albeit the authors did not separate the ascending from the descending phase (Williams et al., 2020). To summarize, while pectoralis major and upper trapezius do not show any difference due to their role, triceps brachii might be excited slightly more in barbell vs. machine overhead press, but only during the descending phase of the front press.

Some considerations and limitations should be acknowledged. In first instance, during a multi-joint exercise, the excitation of the prime movers stringently linked with the excitation of the synergistic muscles involved in that exercise (Saeterbakken et al., 2017). Thus, although each exercise has a series of prime movers, several additional muscles may be involved to manipulate the joint movements, resulting in a compound neuromuscular pattern (Lauver et al., 2016). Indeed, the role of other muscles (e.g., biceps brachii, middle trapezius) was not examined, and this could have deepened the analysis. Moreover, a cross-talk cannot be excluded, although we carefully check the sEMG signal. Second, the present results are reflective of the load and the time under tension selected here and may change should different combinations be used. In this regard, further overhead variations (e.g., using dumbbells or Smith machine) were not examined, and may still be object of future investigations. Third, the present outcomes should not be extended to different populations since the strength training background may influence the neural pattern, and bodybuilders may exhibit unique mass to strength relationship or training velocity. Additionally, the present bodybuilders were not controlled for the use of anabolic steroids, and it is acknowledged that this may mask previous injuries (Pereira et al., 2019), eventually reflecting on the motor pattern. Moreover, the present sample size is low, and conclusions must be drawn with caution. Similarly, men and women may present different responses due to anatomical and mobility differences, and the present results should be extended to women with caution. Last, no kinematic data were recorded, thus bringing more information about the trajectory of the load and the muscle excitation.

Competitive bodybuilders may benefit from the diversification of the overhead press variations in their training routine. When choosing an exercise, practitioners base their choice on the overall neuromuscular and mechanical stimuli that each exercise is supposed to provide. Although there is no established link between the sEMG amplitude and the actual stimulus received by the muscles examined (Vigotsky et al., 2018) especially in term of hypertrophic response (Vigotsky et al., 2022), still the variations examined here may be used to provide diversified stimuli to the muscles surrounding the shoulders. With this in mind, the literature supports that the neural adaptations that underpin the early resistance training-induced increases in strength also imply an augmented recruitment of the agonist muscles (Škarabot et al., 2020), so that the differences reported here for example between front and back overhead may be used to target more specifically the front or back shoulder muscles. Moreover, the greater external load used with the machine press compared to the barbell variations may constitute a greater mechanical stimulus to the shoulder muscles, possibly enhancing the hypertrophic response. Taking it together, competitive bodybuilders could use different overhead press variations, aware that different stimuli to the shoulder muscles will be given.

Another aspect that should be examined in the practice is the safety of performing the overhead press in its back variation, especially with the barbell. A previous study reported higher prevalence of positive response to tests indicating shoulders instability and hyperlaxity when back-to-neck exercises are systematically included in the training routine (Kolber et al., 2013). However, the same study also reported a protective effect of the exercises stimulating the humerus external rotators, hence since back-BMP excites posterior deltoid more than other variations, those outcomes appear contradictory at least (Kolber et al., 2013). Moreover, focusing on the humerus external rotators was shown as protective towards the insurgence of the impingement syndrome (Kolber et al., 2014). Lastly, a more recent study defined it as a “safe exercise” for people with normal trunk stability and ideal shoulder ROM (McKean and Burkett, 2015), and we believe there should not be any reason to exclude it from the training routine. To throw fuel on the fire, back-BMP was shown here more effective in exciting posterior and medial deltoid, therefore should the external humerus rotators be targeted, this might be effectively included in the strength training programmes. Indeed, many overhead sports need for a reinforcement of the external rotators for performance and injury prevention purposes (Cools et al., 2015), and back overhead could be part of the shoulder muscles strengthening routine. In this regard, while acknowledging the importance of specific exercises for the external rotators of the humerus such as external rotations using dumbbells, cables or elastic bands, back-BMP implies the activation and consequently reinforcement of these muscles in a different context, i.e., a complex multi-joint exercise, where more control is needed. Importantly, such a consideration is still valid for sedentary or non-athlete populations, in which a “forward posture” favours a pronounced dorsal kyphosis and internal rotation of the humerus (Heneghan et al., 2018). In such cases, since back overhead forces a simultaneous dorsal lordosis and external rotation of the humerus, once established the safety condition of the exercise (McKean and Burkett, 2015), back-BMP could be implemented in the training starting with light or very light loads (e.g., a broomstick) to be familiarized with the movement, and progressively increase the load. In this regard, it is acknowledged that coaches should be aware of the individual capacities and determine accordingly both load and the movement pattern. Moreover, back-BMP could be an interesting option for individuals who have little time to train, as often occurs with sedentary people. In this case, only a multi-joint exercise could satisfactorily excite the muscular portions of the deltoid without the need to include complementary single-joint exercises for this portion, thus saving time for that session.

## Conclusion

In conclusion, overhead press is effective to stimulate the muscles surrounding the shoulders. More in detail, the back overhead performed with barbell or machine seems to excite the posterior and medial deltoid more than overhead performed with barbell in front or the anterior handgrip of the shoulder press. In contrast, both front presses seem to favour the excitation of pectoralis major and triceps brachii. No difference was observed in anterior deltoid and upper trapezius. Moreover, less stable overhead trajectories when using barbell require greater stabilization than more stable movements as in the cases of shoulder press, and posterior and medial deltoid, and partly anterior deltoid and triceps brachii concurrently act to this purpose.

## Data Availability

The datasets presented in this study can be found in online repositories. The names of the repository/repositories and accession number(s) can be found below: [md5:e8b6c718179c993b0817208341e84130], Zenodo.
